# Bis(2-bromo­benz­yl) tris­ulfide

**DOI:** 10.1107/S1600536809001834

**Published:** 2009-01-23

**Authors:** Suneel. P. Singh, Alan. J. Lough, Adrian. L. Schwan

**Affiliations:** a Department of Chemistry, University of Guelph, 50 Stone Road East, Guelph, Ontario, Canada N1G 2W1; b Department of Chemistry, University of Toronto, 80 St., George Street, Toronto, Ontario, Canada M5S 3H6

## Abstract

The title mol­ecule, C_14_H_12_Br_2_S_3_, lies on a crystallographic twofold rotation axis which bis­ects the S—S—S angle. The dihedral angle between the two symmetry-related benzene rings is 89.91 (9)°. In terms of hybridization principles, the S—C—C angle is slightly larger than expected.

## Related literature

For related literature, see: Haoyun *et al.* (2006 ▶); De Sousa *et al.* (1990 ▶); Johnson *et al.* (1997 ▶); Rys *et al.* (2008 ▶). For a related synthesis see: Banerji & Kalena (1980 ▶); O’Donnell & Schwan (2003 ▶). For a related crystal structure, see: Abu-Yousef *et al.* (2006 ▶).
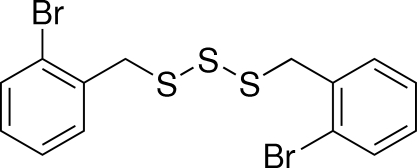


## Experimental

### 

#### Crystal data

C_14_H_12_Br_2_S_3_
*M* *_r_* = 436.24
Orthorhombic, 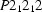

*a* = 12.771 (3) Å
*b* = 13.030 (3) Å
*c* = 4.7635 (10) Å
*V* = 792.7 (3) Å^3^
*Z* = 2
Mo *K*α radiation
μ = 5.49 mm^−1^
*T* = 150 (1) K
0.16 × 0.12 × 0.10 mm

#### Data collection

Nonius KappaCCD diffractometer
Absorption correction: multi-scan (*SORTAV*; Blessing, 1995 ▶) *T*_min_ = 0.378, *T*_max_ = 0.576
5451 measured reflections
1745 independent reflections
1447 reflections with *I* > 2σ(*I*)
*R*_int_ = 0.047

#### Refinement

*R*[*F* ^2^ > 2σ(*F* ^2^)] = 0.034
*wR*(*F* ^2^) = 0.081
*S* = 1.06
1745 reflections
87 parameters
H-atom parameters constrained
Δρ_max_ = 0.32 e Å^−3^
Δρ_min_ = −0.72 e Å^−3^
Absolute structure: Flack (1983 ▶), 659 Friedel pairs
Flack parameter: −0.024 (13)

### 

Data collection: *COLLECT* (Nonius, 2002 ▶); cell refinement: *DENZO-SMN* (Otwinowski & Minor, 1997 ▶); data reduction: *DENZO-SMN*; program(s) used to solve structure: *SIR92* (Altomare *et al.*, 1994 ▶); program(s) used to refine structure: *SHELXTL* (Sheldrick, 2008 ▶); molecular graphics: *PLATON* (Spek, 2003 ▶); software used to prepare material for publication: *SHELXTL*.

## Supplementary Material

Crystal structure: contains datablocks I, global. DOI: 10.1107/S1600536809001834/pv2135sup1.cif

Structure factors: contains datablocks I. DOI: 10.1107/S1600536809001834/pv2135Isup2.hkl

Additional supplementary materials:  crystallographic information; 3D view; checkCIF report

## Figures and Tables

**Table 1:** Selected bond angles (°)

S2^i^—S1—S2	106.21 (9)
C2—C1—S2	114.0 (3)

Symmetry code: (i) 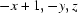.

**Fig. 1.**
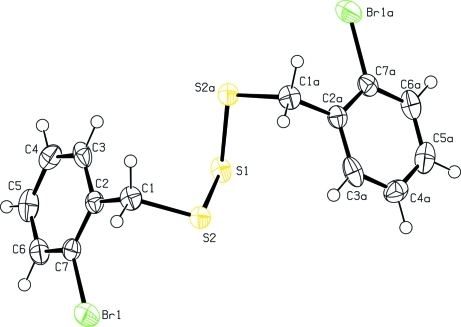
The molecular structure of title compound. All non-H atoms are represented by 30% probability displacement ellipsoids. Atoms labeled with the suffix 'a' are related by the symmetry operator (-x + 1, -y, z).

**Fig. 2.**
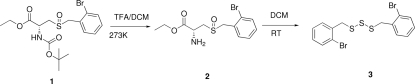
The reaction scheme for the formation of the title compound.

## supplementary crystallographic information

### Comment

Organic sulfides are an attractive class of compounds because of their synthetic and pharmaceutical applications. Dibenzyl trisulfide was isolated from the sub-tropical shrub Petiveria alliacea *L*. (De Sousa *et al.*, 1990; Johnson *et al.*, 1997). Dibenzyl trisulfide dervatives have been synthesized in moderate yield *via* a diimidazolyl sulfide derivative (Banerji & Kalena, 1980). The immunomodulatory activitities, molecular mechanism, anti tumor activities and some other biological activities of dibenzyltrisulfide derivatives have been reported (Haoyun *et al.*, 2006).

In the title molecule (Fig. 1), the bond lengths and bond angles are comparable to those observed in a similar compound (Abu-Yousef *et al.*, 2006).

### Experimental

The N-protected amino acid derivative (1) (Fig. 2) was synthesized by following the described procedure for its benzyl analog (O'Donnell & Schwan, 2003). Compound (1) was reacted with trifluoroacetic acid (20 equiv.) at 273 K for 2 hr to give amino acid derivative (2). The title compound (3) was prepared by stirring (2) in dichloromethane at room temperature in 20% yield and was crystallized by slow evaporation of a dichloromethane/methanol (9:1, *v*/*v*) solution. It should be noted that compound (2) in dichlorometane on standing at room temperature for several days also gave compound (3).

### Refinement

H atoms bonded to C atoms were placed in calculated positions with C—H = 0.95 - 0.99Å and were included in a riding-model approximation with *U*_iso_(H) = 1.2*U*_eq_(C).

### Crystal data

**Table:**

C_14_H_12_Br_2_S_3_	*F*(000) = 428
*M**_r_* = 436.24	*D*_x_ = 1.828 Mg m^−^^3^
Orthorhombic, *P*2_1_2_1_2	Mo *K*α radiation, λ = 0.71073 Å
Hall symbol: P 2 2ab	Cell parameters from 5451 reflections
*a* = 12.771 (3) Å	θ = 3.1–27.5°
*b* = 13.030 (3) Å	µ = 5.49 mm^−^^1^
*c* = 4.7635 (10) Å	*T* = 150 K
*V* = 792.7 (3) Å^3^	Block, colourless
*Z* = 2	0.16 × 0.12 × 0.10 mm

### Data collection

**Table:**

Nonius KappaCCD diffractometer	1745 independent reflections
Radiation source: fine-focus sealed tube	1447 reflections with *I* > 2σ(*I*)
graphite	*R*_int_ = 0.047
Detector resolution: 9 pixels mm^-1^	θ_max_ = 27.5°, θ_min_ = 3.1°
φ scans and ω scans with κ offsets	*h* = −16→16
Absorption correction: multi-scan (*SORTAV*; Blessing, 1995)	*k* = −16→16
*T*_min_ = 0.378, *T*_max_ = 0.576	*l* = −6→6
5451 measured reflections	

### Refinement

**Table:**

Refinement on *F*^2^	Secondary atom site location: difference Fourier map
Least-squares matrix: full	Hydrogen site location: inferred from neighbouring sites
*R*[*F*^2^ > 2σ(*F*^2^)] = 0.034	H-atom parameters constrained
*wR*(*F*^2^) = 0.081	*w* = 1/[σ^2^(*F*_o_^2^) + (0.0416*P*)^2^] where *P* = (*F*_o_^2^ + 2*F*_c_^2^)/3
*S* = 1.06	(Δ/σ)_max_ = 0.001
1745 reflections	Δρ_max_ = 0.32 e Å^−^^3^
87 parameters	Δρ_min_ = −0.72 e Å^−^^3^
0 restraints	Absolute structure: Flack (1983), 659 Friedel pairs
Primary atom site location: structure-invariant direct methods	Flack parameter: −0.024 (13)

### Special details

**Table:**

Geometry. All e.s.d.'s (except the e.s.d. in the dihedral angle between two l.s. planes) are estimated using the full covariance matrix. The cell e.s.d.'s are taken into account individually in the estimation of e.s.d.'s in distances, angles and torsion angles; correlations between e.s.d.'s in cell parameters are only used when they are defined by crystal symmetry. An approximate (isotropic) treatment of cell e.s.d.'s is used for estimating e.s.d.'s involving l.s. planes.
Refinement. Refinement of *F*^2^ against ALL reflections. The weighted *R*-factor *wR* and goodness of fit *S* are based on *F*^2^, conventional *R*-factors *R* are based on *F*, with *F* set to zero for negative *F*^2^. The threshold expression of *F*^2^ > σ(*F*^2^) is used only for calculating *R*-factors(gt) *etc*. and is not relevant to the choice of reflections for refinement. *R*-factors based on *F*^2^ are statistically about twice as large as those based on *F*, and *R*- factors based on ALL data will be even larger.

### Fractional atomic coordinates and isotropic or equivalent isotropic displacement parameters (Å^2^)

**Table:**

	*x*	*y*	*z*	*U*_iso_*/*U*_eq_	
Br1	0.42247 (3)	0.38038 (3)	0.13013 (11)	0.04922 (17)	
S1	0.5000	0.0000	0.2536 (3)	0.0305 (3)	
S2	0.53734 (6)	0.11976 (7)	−0.0036 (2)	0.0319 (2)	
C1	0.4113 (3)	0.1583 (3)	−0.1526 (8)	0.0311 (8)	
H1A	0.4221	0.2196	−0.2718	0.037*	
H1B	0.3853	0.1024	−0.2750	0.037*	
C2	0.3293 (3)	0.1822 (3)	0.0620 (7)	0.0271 (8)	
C3	0.2529 (3)	0.1057 (3)	0.1324 (8)	0.0356 (9)	
H3A	0.2544	0.0412	0.0395	0.043*	
C4	0.1784 (3)	0.1239 (3)	0.3296 (8)	0.0369 (9)	
H4A	0.1282	0.0724	0.3722	0.044*	
C5	0.1750 (3)	0.2175 (3)	0.4693 (9)	0.0433 (11)	
H5A	0.1232	0.2294	0.6086	0.052*	
C6	0.2470 (3)	0.2930 (3)	0.4058 (8)	0.0367 (10)	
H6A	0.2446	0.3572	0.5002	0.044*	
C7	0.3222 (3)	0.2750 (3)	0.2054 (8)	0.0283 (8)	

### Atomic displacement parameters (Å^2^)

**Table:**

	*U*^11^	*U*^22^	*U*^33^	*U*^12^	*U*^13^	*U*^23^
Br1	0.0469 (2)	0.0294 (2)	0.0713 (3)	−0.00040 (19)	−0.0036 (2)	0.0041 (2)
S1	0.0356 (7)	0.0339 (7)	0.0219 (6)	0.0098 (6)	0.000	0.000
S2	0.0286 (4)	0.0303 (4)	0.0369 (5)	0.0012 (4)	0.0002 (4)	−0.0007 (5)
C1	0.0356 (19)	0.0353 (18)	0.0224 (18)	0.0040 (16)	−0.0018 (16)	0.0010 (15)
C2	0.0272 (18)	0.0307 (19)	0.023 (2)	0.0053 (15)	−0.0051 (13)	0.0018 (15)
C3	0.043 (2)	0.037 (2)	0.027 (2)	0.0185 (18)	−0.015 (2)	−0.007 (2)
C4	0.0286 (19)	0.044 (2)	0.038 (2)	−0.0044 (18)	−0.0051 (16)	0.010 (2)
C5	0.033 (2)	0.063 (3)	0.033 (2)	0.016 (2)	0.0022 (18)	0.004 (2)
C6	0.038 (2)	0.041 (2)	0.031 (2)	0.0149 (19)	−0.0079 (19)	−0.0082 (18)
C7	0.0277 (19)	0.0294 (18)	0.028 (2)	0.0042 (14)	−0.0081 (15)	0.0033 (15)

### Geometric parameters (Å, °)

**Table:**

Br1—C7	1.911 (3)	C3—C4	1.358 (5)
S1—S2^i^	2.0403 (13)	C3—H3A	0.9500
S1—S2	2.0403 (13)	C4—C5	1.389 (5)
S2—C1	1.829 (3)	C4—H4A	0.9500
C1—C2	1.496 (5)	C5—C6	1.380 (6)
C1—H1A	0.9900	C5—H5A	0.9500
C1—H1B	0.9900	C6—C7	1.374 (5)
C2—C7	1.392 (5)	C6—H6A	0.9500
C2—C3	1.435 (5)		
			
S2^i^—S1—S2	106.21 (9)	C2—C3—H3A	119.5
C1—S2—S1	103.71 (12)	C3—C4—C5	120.5 (4)
C2—C1—S2	114.0 (3)	C3—C4—H4A	119.8
C2—C1—H1A	108.7	C5—C4—H4A	119.8
S2—C1—H1A	108.7	C6—C5—C4	120.0 (4)
C2—C1—H1B	108.7	C6—C5—H5A	120.0
S2—C1—H1B	108.7	C4—C5—H5A	120.0
H1A—C1—H1B	107.6	C7—C6—C5	119.8 (4)
C7—C2—C3	116.4 (3)	C7—C6—H6A	120.1
C7—C2—C1	124.2 (3)	C5—C6—H6A	120.1
C3—C2—C1	119.4 (3)	C6—C7—C2	122.3 (3)
C4—C3—C2	121.1 (4)	C6—C7—Br1	118.4 (3)
C4—C3—H3A	119.5	C2—C7—Br1	119.2 (3)

Symmetry codes: (i) −*x*+1, −*y*, *z*.
